# Editorial: Continuous Quality Improvement (CQI)—Advancing Understanding of Design, Application, Impact, and Evaluation of CQI Approaches

**DOI:** 10.3389/fpubh.2017.00306

**Published:** 2017-11-23

**Authors:** Ross Bailie, Jodie Bailie, Sarah Larkins, Edward Broughton

**Affiliations:** ^1^The University of Sydney, The University Centre for Rural Health, Lismore, NSW, Australia; ^2^James Cook University, College of Medicine and Dentistry, Townsville, QLD, Australia; ^3^University Research Co., LLC, Chevy Chase, MD, United States

**Keywords:** primary health care, health systems research, continuous quality improvement, Aboriginal and Torres Strait Islander health, building block

**Editorial on the Research Topic**

**Continuous Quality Improvement (CQI)—Advancing Understanding of Design, Application, Impact, and Evaluation of CQI Approaches**

Continuous quality improvement (CQI) approaches are increasingly used to bridge gaps between the evidence base for best practice, what actually happens in practice, and achievement of better population health outcomes. Among a range of quality improvement strategies, CQI is characterized by iterative use of processes to identify quality problems, develop solutions, and implement and evaluate changes. Application of CQI in health care is evolving and evidence of their success continues to emerge (1–3).

Through the Research Topic, “Continuous Quality Improvement (CQI)—Advancing Understanding of Design, Application, Impact, and Evaluation of CQI approaches,” we aimed to aggregate knowledge of useful approaches to tailoring CQI approaches for different contexts, and for implementation, scale-up and evaluation of CQI interventions/programs. This Research Topic has attracted seven original research reports and three “perspectives” papers. Thirty-six authors have contributed from eighteen research organizations, universities, and policy and service delivery organizations. All original research articles and one perspective paper come from the Australian Audit and Best Practice for Chronic Disease (ABCD) National Research Partnership (“ABCD Partnership”) in Indigenous primary healthcare settings (4–6). To some extent, this reflects the interests and connections of two of the Topic Editors, who were lead investigators on the ABCD Partnership. This Partnership has made a prominent contribution to original research on CQI in primary healthcare internationally, with over 50 papers published in the peer-reviewed literature over the past 10 years.

As most articles in this Research Topic arise from the ABCD Partnership, a brief overview of the program provides a useful backdrop. The program originated in 2002 in the Top End of the Northern Territory in Australia, and built on substantial prior research and evaluation of CQI methods in Indigenous primary healthcare. With substantial growth and enthusiastic support from service providers and researchers around Australia, the ABCD Partnership has focused since 2010 on exploring clinical performance variation, examining strategies for improving primary care, and working with health service staff, management and policy makers to enhance effective implementation of successful strategies (4). By the end of 2014, the ABCD Partnership had generated the largest and most comprehensive dataset on quality of care in Australian Indigenous primary healthcare settings. The Partnership’s work is being extended through the Centre of Research Excellence in Integrated Quality Improvement (6).

Several research papers included in this Research Topic illustrate consistent findings of wide variation in adherence to clinical best-practice guidelines between health centers (Bailie et al.; Burnett et al.; Matthews et al.). The papers also show variation among different aspects of care, with relatively good delivery of some modes of care [Bailie et al.; (7)] and poor delivery of others—such as follow-up of abnormal clinical or laboratory findings. These findings are evident in eye care (Burnett et al.), general preventive clinical care (Bailie et al.), and in absolute cardiovascular risk assessment (Matthews et al.; Vasant et al.). The findings are consistent with other ABCD-related publications on diabetes care (8), preventive health (9), maternal care (10), child health (11), rheumatic heart disease (12), and sexual health (13).

Systems to support good clinical care are explored by Woods et al. in five primary healthcare centers that were identified through ABCD data as achieving substantially greater improvement than others over successive CQI cycles. Attention to understanding and improving systems was shown to be vital to the improvements in clinical care achieved by these health centers. Improved staffing and commitment to working in the community were standout aspects of health center systems that underpinned improvements in clinical care.

On a wider scale, engagement by primary healthcare services in the ABCD Partnership has enabled assessment of system functioning at district, regional, state, and national levels, as reflected in stakeholders’ perceptions of barriers and enablers to addressing gaps in chronic illness care and child health, and identifying drivers for improvement (Bailie et al.). Primary drivers included staff capability, availability and use of information systems and decision support tools, embedding of CQI processes, and community engagement. We have also shown how consistent and sustained policy and infrastructure support for CQI enables large-scale and ongoing improvements in quality of care (3).

Commitment of the ABCD team to promoting effective use of CQI data is reflected in one “perspective” paper, which describes a theory-informed cyclical interactive dissemination strategy (Laycock et al.). Concurrent developmental evaluation provides a mechanism for learning and refinement over successive cycles (14).

The other two perspective articles (not specifically from the ABCD program) highlight the role of facilitation in CQI and the potential for application of CQI in health professional education. The emerging evidence on facilitation as a vital tool for effective CQI should guide resourcing and approaches to CQI (Harvey and Lynch). The approach builds on the humanistic principles of modern CQI methods—participation, engagement, shared decision-making, enabling others, and tailoring to context. The framework for CQI approaches to health professional education described by Clithero et al. directly addresses a critical need for innovative approaches to health workforce development that will strengthen community engagement and embed CQI principles into health system functioning. The scale and scope of need in workforce development is strongly evident in findings of the ABCD program.

Importantly, CQI methods are proving useful in assessing and potentially improving delivery of evidence-based health promotion practices (Percival et al.). Percival’s experience in this field highlights the health facility and wider system challenges facing effective implementation of CQI methods. In health promotion these barriers include low priority given to health promotion in the face of heavy demands for acute clinical care. This work in health promotion complements other research on applying CQI to social determinants of health more broadly (15), including community food supply (16), housing (17), and education (18).

The publications in this special issue address many of the “building blocks” of high performing primary care described by Bodenheimer and colleagues in the US; namely, four foundational components (engaged leadership, data-driven improvement, empanelment, and team-based care) that are vital to facilitate the implementation of the other six elements (patient-team partnership, population management, continuity of care, prompt access to care, comprehensiveness, and care coordination) (19). They are also relevant to Australian based work on clinical microsystems and development of CQI tools for mainstream general practice, such as the Primary Care-Practice Improvement Tool (with similar components to the ABCD systems assessment tool) (20).

Continuous quality improvement is vital to improving health outcomes through system strengthening. We anticipate substantial future development of CQI methods. By late 2017, there had been over 20,000 views of this Research Topic, and many articles have already been cited in peer-review manuscripts. Further research on CQI in primary healthcare would be well guided by a systematic scoping review of literature summarizing empirical research on current knowledge in the field, and identifying key knowledge gaps.

## Author Contributions

RB wrote the first draft. JB has revised content and structure. SL and EB reviewed and edited subsequent drafts. All authors have approved the final version of the manuscript for publication.

## Conflict of Interest Statement

The authors declare that the research was conducted in the absence of any commercial or financial relationships that could be construed as a potential conflict of interest. RB was the chief investigator on the ABCD National Research Partnership and is the chief investigator on the Centre of Research Excellence in Integrated Quality Improvement. All papers published in the Research Topic received peer review from members of the Frontiers in Public Health Policy panel of reviewers who were independent of named authors on any given article published in this volume, consistent with the journal policy on conflict-of-interest.

## Abbreviations

ABCD, audit and best practice for chronic disease; CQI, continuous quality improvement.
